# NXT1, a Novel Influenza A NP Binding Protein, Promotes the Nuclear Export of NP via a CRM1-Dependent Pathway

**DOI:** 10.3390/v8080209

**Published:** 2016-07-28

**Authors:** Nopporn Chutiwitoonchai, Yoko Aida

**Affiliations:** Viral Infectious Diseases Unit, RIKEN, Wako, Saitama 351-0198, Japan; nopporn.chutiwitoonchai@riken.jp

**Keywords:** Influenza A virus, NP, NXT1, CRM1, nuclear export

## Abstract

Influenza remains a serious worldwide public health problem. After infection, viral genomic RNA is replicated in the nucleus and packed into viral ribonucleoprotein, which will then be exported to the cytoplasm via a cellular chromosome region maintenance 1 (CRM1)-dependent pathway for further assembly and budding. However, the nuclear export mechanism of influenza virus remains controversial. Here, we identify cellular nuclear transport factor 2 (NTF2)-like export protein 1 (NXT1) as a novel binding partner of nucleoprotein (NP) that stimulates NP-mediated nuclear export via the CRM1-dependent pathway. NXT1-knockdown cells exhibit decreased viral replication kinetics and nuclear accumulated viral RNA and NP. By contrast, NXT1 overexpression promotes nuclear export of NP in a CRM1-dependent manner. Pull-down assays suggest the formation of an NXT1, NP, and CRM1 complex, and demonstrate that NXT1 binds to the C-terminal region of NP. These findings reveal a distinct mechanism for nuclear export of the influenza virus and identify the NXT1/NP interaction as a potential target for antiviral drug development.

## 1. Introduction

Influenza virus infection has resulted in several serious pandemics e.g., Spanish influenza (H1N1) in 1918 [1] and the swine influenza (H1N1) in 2009 [2]. In addition, human infections with a new avian influenza A (H7N9) subtype were reported in China in 2013 [3]. A seasonal influenza vaccine is available but must be reformulated yearly. Few antiviral drugs have been approved by the U.S. Food and Drug Administration, including oseltamivir, zanamivir, and peramivir, all of which target viral neuraminidase (NA) to inhibit viral release from host cells, and the development and transmission of oseltamivir- or zanamivir-resistant viruses has been reported [4,5,6,7]. Thus, there is an urgent need for the identification of novel potential targets and the development of new antiviral drugs.

Viral nucleoprotein (NP) plays multiple roles in viral lifecycle, including nuclear import/export of viral ribonucleoprotein (vRNP) and transcription/replication of the viral genome [8,9,10]. Targeting at the unconventional nuclear localization signal (NLS) of NP by mycalamide analogs results in a reduction of viral replication [11]. In addition, inhibition of NP nuclear localization by nucleozin and its analogs potentially decreases in vitro and in vivo viral replication [12,13]. Our previous comparative study of influenza nuclear export signals (NESs), including matrix 1 (M1)-NES, non-structural 1 (NS1)-NES, nuclear export protein (NEP)-NES1, NEP-NES2, NP-NES1, NP-NES2, and NP-NES3, identified NP-NES3 as a promising antiviral drug target [14]. Moreover, the NP-NES3 inhibitors RK424 and DP2392-E10 showed a potent inhibitory effect against viral replication [15,16]. This evidence indicates NP as an attractive target for antiviral drug development.

Cellular nuclear export machinery is required for viral replication, since viral RNA replication and vRNP assembly occur in the nucleus and vRNPs must be exported to the cytoplasm for assembly and budding at the plasma membrane. The vRNP complex comprises viral genomic RNA, viral polymerase complex (polymerase acidic protein (PA), polymerase basic protein 1 (PB1), and polymerase basic protein 2 (PB2)), and a multimeric NP. Nuclear export of vRNPs is proposed to be mediated by a cellular chromosome region maintenance 1 (CRM1) pathway in which the association of viral M1 and NEP link vRNP to CRM1 [17,18]. In addition, an additional interaction between NEP and viral polymerase of vRNP was identified and proposed to stabilize the M1/vRNP association [19]. Targeting of CRM1-mediated nuclear export by leptomycin B (LMB), an irreversible inhibitor that binds covalently to CRM1 [20], inhibited influenza vRNP nuclear export [21]. In addition, verdinixor, a novel CRM1 inhibitor that forms a slowly-reversible covalent bond to CRM1, showed an inhibitory effect against H1N1, H5N1, and H7N9 viruses [22]. However, LMB and verdinixor target CRM1 at the leucine-rich groove that is the NES binding site, which is also required for the function of other cellular NES-containing proteins. Thus, the identification of virus-specific targets is necessary for the development of antiviral drugs with minimal cytotoxicity or side effects. 

Several novel cellular factors involved in nuclear export of the influenza virus have been identified. Heat shock cognate 70 (Hsc70) was reported to bind viral M1 and mediate nuclear export of the vRNP/M1/NEP complex [23,24]. Chromodomain-helicase-DNA-binding protein 3 (CHD3) was shown to interact with NEP-NES1 to localize NEP/CRM1 to dense chromatin and facilitate vRNP nuclear export via a CRM1-dependent pathway [25]. Knockdown of nucleoporin 62 kDa (NUP62), a component of the nuclear pore complex, inhibited the nuclear export of viral mRNA/viral RNA (vRNA) and reduced viral replication [26,27]. In addition, interaction between NUP62 and viral polymerase was identified [27]. Nucleoporin 98 kDa (NUP98) was reported to interact with NEP via its N-terminal phenylalanine-glycine (FG)-repeat domain, and overexpression of dominant-negative NUP98 decreased viral replication [28], whereas knockdown of NUP98 has no effect on vRNA nuclear export or replication [26]. Cellular mRNA nuclear export factors, including transporter associated with antigen processing (TAP) (or nuclear RNA export factor 1 (NXF1)), nuclear transport factor 2 (NTF2)-like export protein 1 (NXT1 or p15), ribonucleic acid export 1 (Rae1), and E1B-55kDa-associated protein 5 (E1B-AP5), have been reported to bind with viral NS1 [29], and interaction of viral polymerase with cellular TAP, RNA binding motif protein 15B (RMB15B), and DEAD-Box Helicase 19B (DDX19B) was identified [27]. However, the precise role of these nuclear export factors in the molecular mechanism of viral replication has not been established, and this will be necessary for a comprehensive understanding of influenza virus nuclear export and identification of potential targets for antiviral drug development. 

In this study, we selected a number of host factors reported to be involved with cellular nuclear export function and influenza virus replication, and subjected them to affinity binding assays with NP. Our results identified NXT1 as an NP binding partner. NXT1 interacts with NP and CRM1 to stimulate NP nuclear export via a CRM1-dependent pathway and to promote viral replication. 

## 2. Materials and Methods

### 2.1. Cells, Viruses, and Transfections

HeLa, human embryonic kidney (HEK) 293T, Madin-Darby canine kidney (MDCK), and A549 cells were maintained in Dulbecco’s Modified Eagle’s Medium (DMEM, Gibco, Beijing, China) supplemented with 10% fetal bovine serum (FBS, Gibco and HyClone Laboratories, Logan, UT, USA) in a 5% CO_2_ incubator at 37 °C. Infectious influenza A/WSN/33 (H1N1) virus was produced by reverse genetics, as described previously [14]. FuGENE HD (Promega, Fitchburg, WI, USA) and Lipofectamine 2000 (Invitrogen, Waltham, MA, USA) were used for plasmid transfection and Lipofectamine RNAiMAX Reagent (Invitrogen) was used for siRNA transfection throughout the study.

### 2.2. Plasmid Construction

Plasmids for the reverse genetic generation of influenza A/WSN/33 (H1N1) viruses, including PA/pHH21, PB1/pHH21, PB2/pHH21, NP/pHH21, M/pHH21, NS/pHH21, hemagglutinin (HA)/pHH21, NA/pHH21, PA/pCAGGS, PB1/pCAGGS, PB2/pCAGGS, and NP/pCAGGS [30], were a gift from Yoshihiro Kawaoka (Institute of Medical Science, University of Tokyo, Tokyo, Japan).

The NP-FLAG/pCAGGS plasmid was constructed as described previously [14]. All of the insert sequences were amplified by KOD Plus DNA polymerase (Toyobo, Osaka, Japan), digested with *Xho*I/*Not*I, and sub-cloned into the *Xho*I/*Not*I sites of the modified pCAGGS plasmid [31]. N-terminal partial NP was constructed by amplification of the NP/pCAGGS plasmid using the forward primer 5′-AAACTCGAGACCATGGCGACCAAAGGCACC-3′ with the reverse primer 5′-TTTGCGGCCGCTTACTTGTCATCGTCGTCCTTGTAATCACTTGATTCCATAGTCTC-3′ for NP 1-377 FLAG, the reverse primer 5′-TTTGCGGCCGCTTACTTGTCATCGTCGTCCTTGTAATCATTCCGGCTCTCTCTCAC-3′ for NP 1-247 FLAG, or the reverse primer 5′-CGTCATCCTTGTAATCTCCTGAATCCATTCCTGTGCGAAC-3′ and second reverse primer 5′-TTTGCGGCCGCTTACTTATCGTCGTCATCCTTGTAATCTC-3′ for NP1-160 FLAG. 

NP-NES1 FLAG/pCAGGS (F39A/I41A), NP-NES2 FLAG/pCAGGS (M189A/M191A), and NP-NES3 FLAG/pCAGGS (L264A/L266A) were constructed by amplification of the NP sequence from the NP-NES1/pCAGGS, NP-NES2/pCAGGS, and NP-NES3/pCAGGS plasmids, respectively [14], with the forward primer 5′-AAACTCGAGACCATGGCGACCAAAGGCACC-3′ and the reverse primer 5′-CGTCGTCCTTGTAATCTCCTGAATTGTCGTACTCCTCTG-3′, followed by re-amplification with the extended reverse primer 5′-TTTGCGGCCGCTTACTTGTCATCGTCGTCCTTGTAATCTC-3′. 

The FLAG-NS1/pCAGGS plasmid was constructed using the forward primer 5′-ACTCGAGATGGATTACAAGGATGACGACGATAAGGATCCAAACACTGTGTCA-3′ and the reverse primer 5′-AAAAGCGGCCGCTTACTATCAAACTTCTGACCTAAT-3′ with the template NS/pHH21 plasmid.

The NXT1 and CRM1 plasmids were generated from the total cDNA of HeLa cells. HA-NXT1/pCAGGS was constructed using the forward primer 5′-AAACTCGAGATGTACCCATACGATGTTCCAGATTACGCTGCATCTGTGGATTTCAAG-3′ and the reverse primer 5′-AAAAGCGG CCGCTTATCACTAGCTGGCCCAGTCCTG-3′, while FLAG-NXT1/pCAGGS was constructed using the forward primer 5′-GGATGACGACGATAAGGGCAGCGGCGCATCTGTGGATTTCAAG-3′ and the reverse primer 5′-AAAAGCGGCCGCTTATCACTAGCTGGCCCAGTCCTG-3′, followed by re-amplification with the extended forward primer 5′-AAAACTCGAGACCATGGATTACAAGGATGACGACGATAAGGG-3′. 

The CRM1-HA/pCAGGS plasmid was prepared as described previously [14], while FLAG-CRM1/pCAGGS was constructed by amplification with the forward primer 5′-GGATGACGACGATAAGGGCAGCGGCCCAGCAATTATGACAATG-3′ and the reverse primer 5′-AAAAGCGGCCGCTTAATCACACATTTCTTC-3′, followed by re-amplification with the extended forward primer 5′-AAAACTCGAGACCATGGATTACAAGGATGACGACGATAAGGG-3′. 

All constructed plasmids were checked by DNA sequencing with the BigDye Terminator v.3.1 Cycle Sequencing Kit (Applied Biosystems, Waltham, MA, USA).

### 2.3. Pull-down and Immunoprecipitation Assays

Protein-immobilized (NP-FLAG, FLAG-NS1, HA-NXT1, CRM1-HA, or FLAG and HA only as a control) agarose beads (Anti-FLAG^®^ M2 affinity gel or Monoclonal anti-HA-agarose antibody, both from Sigma-Aldrich, Saint Louis, MO, USA) and purified proteins (NP-FLAG and FLAG-CRM1) for pull-down assay were prepared from transfected HEK293T cells as described previously [14]. 

Pull-down with HeLa whole cell lysate was performed by lysing 7 × 10^6^ HeLa cells with 500 µL of radioimmunoprecipitation assay (RIPA) buffer (50 mM Tris-HCl/pH 8.0, 150 mM NaCl, 1% Nonidet^®^ P-40 (NP-40, Nacalai Tesque, Kyoto, Japan), 0.1% sodium dodecyl sulphate (SDS, Sigma-Aldrich, Saint Louis, MO, USA), and 0.5% sodium deoxycholate (Sigma-Aldrich)) plus a complete protease inhibitor cocktail (Roche, Mannheim, Germany), on ice for 30 min. The cell lysate was clarified by centrifugation at 15,000× *g* and 30 µg of the clear lysate was co-incubated with NP-FLAG- or FLAG-NS1-immobilized agarose beads (Anti-FLAG^®^ M2 affinity gel, Sigma-Aldrich) (15 µL of 50% slurry) in 500 µL of bind/wash buffer-I (20 mM 4-(2-hydroxyethyl)-1-piperazineethanesulfonic acid (HEPES, Sigma-Aldrich)/pH 7.4, 110 mM potassium acetate, 2 mM magnesium chloride, and 0.1% Tween 20 (Nacalai Tesque)) plus protease inhibitor cocktail (Roche) overnight at 4 °C with rotation. The beads were collected by centrifugation at 500× *g*, at 4 °C for 2 min and washed five times with the bind/wash buffer-I. After removing all supernatant, the beads were resuspended in SDS sample buffer (60 mM Tris-HCl/pH 6.8, 2% SDS, 10% glycerol (Sigma-Aldrich), 0.01% bromophenol blue, 1.25% β-mercaptoethanol (Sigma-Aldrich)) and boiled for 5 min before western blot (WB) analysis. 

**Figure 1:**
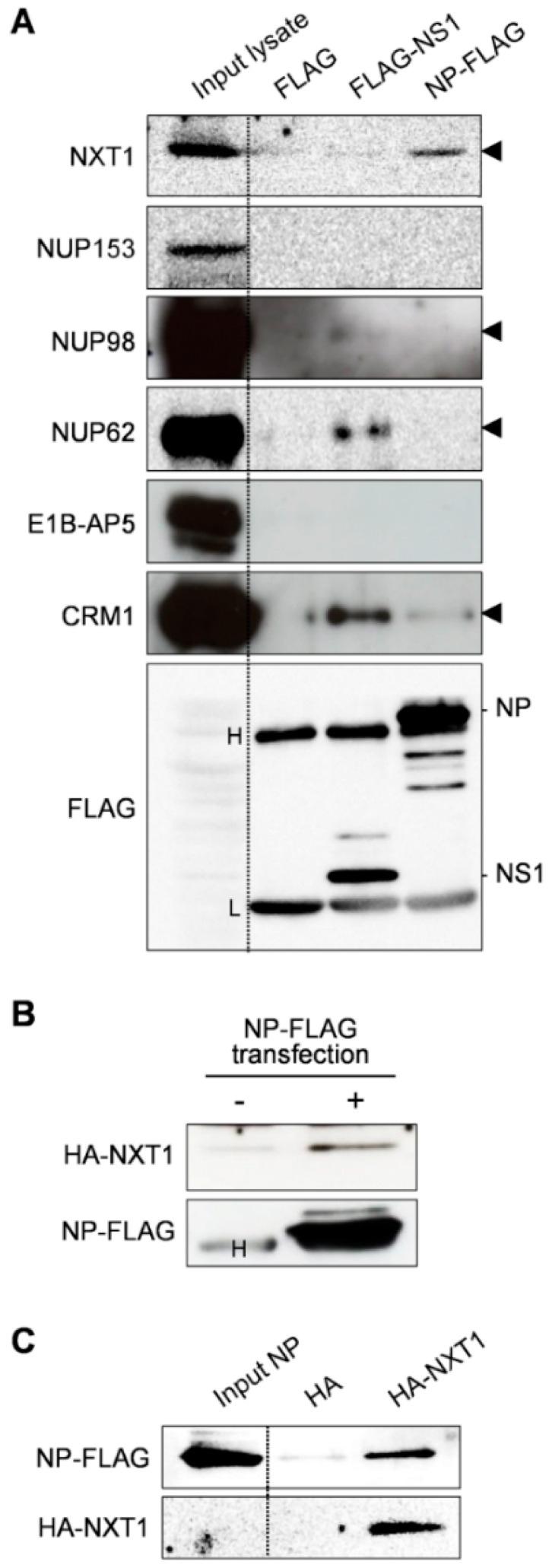
Identification of nuclear transport factor 2 (NTF2)-like export protein 1 (NXT1) as a binding partner of influenza nucleoprotein (NP). (**A**) NP-FLAG or FLAG- non-structural 1 (NS1) protein was immobilized on anti-FLAG agarose beads and co-incubated with HeLa whole cell lysate overnight at 4 °C. The beads were collected, washed, and subjected to western blot (WB) analysis with anti-NXT1, anti-NUP153, anti-NUP98, anti-NUP62, anti-E1B-AP5, anti-CRM1, or anti-FLAG antibodies. Data shown are the results of one representative experiment from two independent trials. Black arrowheads indicate specific binding. H and L indicate the heavy and light chains of the agarose-conjugated anti-FLAG antibody; (**B**) Human embryonic kidney (HEK293T) cells were transfected with NP-FLAG/pCAGGS and HA-NXT1/pCAGGS plasmids for 48 h, lysed, and incubated with anti-FLAG agarose beads overnight at 4 °C. The agarose beads were collected, washed, and subjected to WB analysis with anti- hemagglutinin (HA) and anti-FLAG antibodies. Data shown are the results of one representative experiment from two independent trials; (**C**) HA-NXT1 protein was immobilized on anti-HA agarose beads and co-incubated with purified NP-FLAG protein overnight at 4 °C. The beads were collected, washed, and subjected to WB analysis with anti-FLAG and anti-HA antibodies. Data shown are the results of one representative experiment from three independent trials.

Pull-down with purified protein was performed by co-incubation of protein-immobilized agarose beads (10 µL of 50% slurry) and 1 µg of purified protein (see Figure 1, Figure 6 and Figure 7) in 500 µL of bind/wash buffer-II (25 mM Tris-HCl/pH 7.4, 150 mM NaCl, 1 mM ethylenediaminetetraacetic acid (EDTA, Sigma-Aldrich), 1% NP-40, and 5% glycerol) with protease inhibitor cocktail (Roche) overnight at 4 °C with rotation. The beads were collected, washed with bind/wash buffer-II, and prepared as described above for WB analysis.

Immunoprecipitation was performed by transfection of HA-NXT1/pCAGGS either without/with NP-FLAG/pCAGGS into HEK293T cells for 48 h. The cells were collected and lysed with RIPA buffer plus protease inhibitor cocktail for 30 min on ice. Cell debris was removed by centrifugation at 20,400× *g* at 4 °C for 15 min and the lysate supernatant was collected. A total of 100 µg of lysate was incubated with 20 µL of anti-FLAG M2 affinity gel (Sigma-Aldrich) in 500 µL of bind/wash buffer-I overnight at 4 °C with rotation. The beads were collected by centrifugation at 500× *g*, 4 °C for 2 min, washed with bind/wash buffer-I, and prepared as described above for WB analysis.

### 2.4. Western Blot Analysis

SDS-polyacrylamide gel electrophoresis and WB were performed as described previously [14]. Antibodies used for analysis were anti-NXT1 rabbit polyclonal antibody (pAb) (Abnova, Taipei, Taiwan), anti-CRM1 mouse monoclonal antibody (mAb) (BD Biosciences, San Jose, CA, USA), anti-NUP153 rat mAb (Santa Cruz Biotechnology, Santa Cruz, CA, USA), anti-NUP98 rat mAb (Abcam, Cambridge, UK), anti-NUP62 mouse mAb (BD Biosciences), anti-E1B-AP5 (or hnRNP UL1) mouse mAb (Santa Cruz Biotechnology), anti-FLAG M2 mouse mAb (Sigma-Aldrich, Saint Louis, MO, USA), anti-FLAG rabbit mAb (Sigma-Aldrich), anti-HA mouse mAb (Medical and Biological Laboratories, Nagoya, Japan), anti-β-actin mouse mAb (Sigma-Aldrich), anti-WSN (influenza A/WSN/33) rabbit pAb (a gift from Dr. Kazufumi Shimizu, Nihon University School of Medicine, Tokyo, Japan), horseradish-peroxidase (HRP)-conjugated goat anti-mouse IgG (Amersham Biosciences, Uppsala, Sweden), or HRP-conjugated goat anti-rabbit IgG (Amersham Biosciences). The intensity of NP bands was quantitated by ImageJ 1.50 software (National Institutes of Health, Bethesda, MD, USA).

### 2.5. NXT1 Knockdown

Stealth RNAi™ siRNA (Invitrogen) targeting the NXT1 gene (5′-GAGTTTGTCAATGTCTACTACACCA-3′) was designed by BLOCK-iT RNAi Designer (Invitrogen) and ordered from Invitrogen. A control siRNA was Stealth RNAi siRNA Negative Control Med GC (Invitrogen). A mixture of siRNA for transfection was prepared as described below. 

### 2.6. Cell Viability

MDCK or A549 (3 × 10^4^) cells were seeded in a 96-well plate together with control or NXT1 siRNA (0.075 µL of 10 nM siRNA and 0.375 µL of RNAiMax in 25 µL of Opti-MEM™ (Gibco, Grand Island, NY, USA)) and cultured for 24 h or 48 h. The cells were washed with phosphate buffered saline (PBS) and cultured with 100 µL of fresh DMEM plus 10 µL of Premix Water Soluble Tetrazolium Salt (WST-1, Takara, Shiga, Japan) at 37 °C for 20 min. The optical density 450 nm (OD450) was measured by Wallac 1420 Victor 2 microplate reader (Perkin Elmer, Waltham, MA, USA).

### 2.7. Virus Replication

**Figure 2:**
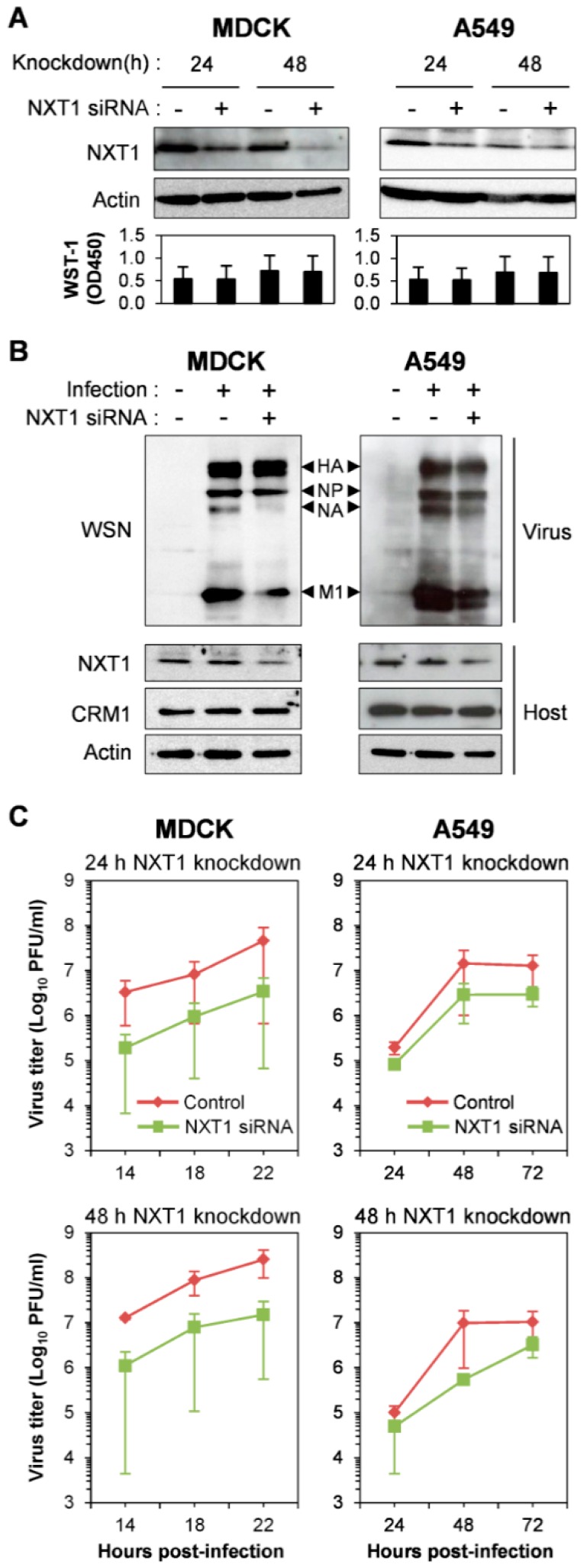
NXT1 knockdown reduces viral replication kinetics. (**A**) Madin-Darby canine kidney (MDCK) or A549 cells were transfected with control or NXT1 siRNA for 24 or 48 h. The cells were subjected to WB analysis with anti-NXT1 and anti-β-actin antibodies (upper panel) or water-soluble tetrazolium (WST-1) assay (lower panel). Data shown are the results from two independent experiments and the mean values. (**B**) MDCK or A549 cells were transfected with control or NXT1 siRNA for 24 h and infected with influenza A/WSN/33 virus at a multiplicity of infection (MOI) = 0.0001 for 18 h and 48 h, respectively. The cells were then collected, lysed, and subjected to WB analysis with anti-WSN, anti-NXT1, anti-CRM1, and anti-β-actin antibodies. (**C**) MDCK or A549 cells were transfected with control or NXT1 siRNA for 24 h or 48 h, and infected with influenza A/WSN/33 virus at MOI = 0.0001. Supernatants were collected at 14, 18, and 22 h post-infection (hpi) from MDCK cells, or at 24, 48, and 72 hpi from A549 cells for virus titration by plaque assay. Viral replication kinetics were plotted for virus titer and time post-infection. Data shown are the results from two independent experiments (error bars) and the mean values.

MDCK (2.5 × 10^5^) or A549 (5 × 10^5^) cells were seeded in a six-well plate together with control or NXT1 siRNA (1.5 µL of 10 nM siRNA and 7.5 µL of RNAiMax in 500 µL of Opti-MEM) and cultured for 24 h or 48 h. The cells were infected with influenza A/WSN/33 virus at a multiplicity of infection (MOI) = 0.0001, as described previously [14]. The infected MDCK cells were cultured in DMEM supplemented with 10% FBS, with 1 µg/ml of tosylsulfonyl phenylalanyl chloromethyl ketone-treated trypsin (TPCK-trypsin; Worthington Biochemical Corporation, Lakewood, NJ, USA) to enhance viral replication efficiency while the infected A549 cells were cultured without TPCK-trypsin since the cells are sensitive to trypsin. Culture medium containing virus was collected at the indicated time points (see Figure 2) and titrated for virus concentration by plaque assay, as described previously [14]. In order to check viral protein expressions, the 24 h NXT1-knockdown MDCK cells at 18 h post-infection (hpi) and A549 cells at 48 hpi were collected, lysed with NET buffer (10 mM Tris-HCl/pH 7.4, 150 mM NaCl, 1 mM EDTA, and 1% NP-40) plus protease inhibitor cocktail, and boiled with SDS sample buffer for WB analysis of viral and cellular proteins (see Figure 2).

### 2.8. Immunofluorescence Staining

MDCK cells (4 × 10^4^) were seeded on cover glass in a 12-well plate together with control or NXT1 siRNA (0.6 µL of 10 nM siRNA and 3 µL of RNAiMax in 200 µL of Opti-MEM) and cultured for 48 h. The cells were infected with influenza A/WSN/33 virus at MOI = 2 for 2, 4, or 6 h before immunofluorescence staining. 

HeLa cells (4 × 10^4^) were seeded overnight on cover glasses in a 12-well plate and transfected with 1 µg of NP-FLAG/pCAGGS either without/with 1 µg of FLAG-NXT1/pCAGGS or 1 µg of FLAG-CRM1/pCAGGS plasmids. At 36 h after transfection, 10 nM of LMB (Sigma-Aldrich) was added and the cells were cultured for 12 h before immunofluorescence staining. 

Immunofluorescence staining was performed as described previously [14] with the following antibodies: anti-NP mouse mAb (Santa Cruz Biotechnology), anti-M1 mouse mAb (Abcam), anti-NEP rabbit pAb (GeneTex, Irvine, CA, USA), anti-FLAG rabbit mAb (Sigma-Aldrich), Alexa Fluor 594 goat anti-mouse IgG (Invitrogen), Alexa Fluor 594 goat anti-rabbit IgG (Invitrogen), and Alexa Fluor 488 goat anti-rabbit IgG (Invitrogen). Nuclei were stained with Hoechst 33342 (ImmunoChemistry Technologies LLC, Bloomington, MN, USA) at room temperature for 5 min. Intracellular localization of the stained proteins was visualized using a laser scanning confocal fluorescence microscope (IX81-FV1000-D/FLUOVIEW System, Olympus, Melville, NY, USA). Nuclear accumulation of NP, M1, or NEP in the NXT1-knockdown/infected MDCK cells was quantified by measuring the mean fluorescence intensity of Alexa Fluor 594 in the nuclear area using ImageJ v.1.50 software and normalized with the mean fluorescence intensity of Hoechst 33342. Counting of cells with nuclear or cytoplasmic localization of NP in the LMB-treatment experiment was performed by ImageJ v.1.50 software and calculated as % of total cell count.

### 2.9. Fluorescence In Situ Hybridization (FISH)

MDCK cells (1 × 10^5^) were seeded on cover glasses in a 12-well plate together with control or NXT1 siRNA (0.6 µL of 10 nM siRNA and 3 µL of RNAiMax in 200 µL of Opti-MEM) and cultured for 48 h before being infected with influenza A/WSN/33 virus at MOI = 10. At 4 and 6 hpi, the cells were analyzed by FISH, as described previously [16,32,33]. Briefly, the cells were washed with 5 mM MgCl_2_/PBS, fixed with 4% paraformaldehyde, and permeabilized with 0.5% Triton X-100/PBS before incubation with 2x saline sodium citrate buffer (SSC, Sigma-Aldrich) plus 10% formamide. The cells were then incubated with 2 µM of Stellaris RNA FISH Probes (Biosearch Technologies, Petaluma, CA, USA; 48 oligo mixture of each 20-mer single-strand DNA conjugated to Quasar 670) targeting viral PB2 segment RNA in hybridization buffer (10% dextran, 2 mM vanadyl ribonucleoside complexes, 0.02% bovine serum albumin (BSA), 50 mg *Escherichia coli* tRNA, 2x SSC, and 10% formamide) at 37 °C for 16 h. The cells were then washed and stained with Hoechst 33342, followed by immunofluorescence staining with anti-NP mouse mAb (Santa Cruz Biotechnology) and Alexa Fluor 488 goat anti-mouse IgG (Invitrogen). Counting of cells with nuclear localization of PB2-RNA was performed by ImageJ v.1.50 software and calculated as a percent of the total cell count.

## 3. Results

### 3.1. Identification of NXT1 as a Binding Partner of Influenza NP

To identify host factors that play a role in NP-mediated nuclear export of the influenza virus, we collected a list of candidate host factors that have been reported to be involved in nuclear export function and influenza virus replication, including NXT1, nucleoporin 153 (NUP153), NUP98, NUP62, and E1B-AP5 [26,29,34]. To detect specific binding to NP, pull-down assays were carried out by incubating whole cell lysates of HeLa cells with NP-FLAG immobilized on agarose beads. After WB analysis with specific antibodies for each of the candidate host factors, we detected binding between NP and NXT1 (Figure 1A). Influenza NS1, which is reported to bind to NXT1, E1B-AP5, and NUP98 [29], was used as a positive control. However, we could not detect the interaction of NS1 with NXT1, E1B-AP5, or NUP98, which may be due to a difference between assay systems, such as NS1 construction (90% identities), NS1 protein expression/purification quality, or the pull-down efficiency of the tagged protein. Specific interaction between NP and CRM1 was reported previously [14], and was used to verify our NP construction and pull-down assay system (Figure 1A). We further verified the binding of NP/NXT1 by immunoprecipitation using anti-FLAG agarose beads with NP-FLAG and HA-NXT1 transfected HEK293T lysate. An NXT1 band was detected only in the lysate of cells transfected with NP-FLAG, indicating specific binding of NP and NXT1 (Figure 1B). Finally, we performed a pull-down assay using HA-NXT1-agarose beads and purified NP-FLAG protein to confirm the NP/NXT1 binding (Figure 1C). Together, these findings demonstrate that NP binds specifically to NXT1 protein.

### 3.2. NXT1 Promotes Replication of Influenza A Virus

We used siRNA knockdown of NXT1 to determine the function of NXT1 in influenza replication. Since NXT1 plays a role in the nuclear export of cellular mRNA [35,36,37], tRNA, and U1 small nuclear RNA (snRNA) [38], the host cells for infection, MDCK and A549, were partially knocked-down with NXT1 siRNA and showed a decrease of NXT1 expression without an effect on the cell viability (Figure 2A). In addition, this NXT1 knockdown system did not affect cellular protein expression, at least of β-actin and CRM1, in the infected cells (Figure 2B). The short-term, 24 h NXT1-knockdown cells were used for infection and WB analysis to avoid the effect of severe NXT1 depletion which might increase the cytopathic effect of virus infection and cause reduced detection of cellular proteins. Viral replication kinetics were then assessed at different time points post-infection and showed decreased rates of viral replication in both NXT1-knockdown MDCK and A549 cells (Figure 2C). These results indicate that NXT1 is necessary for promoting viral production and replication. 

**Figure 3:**
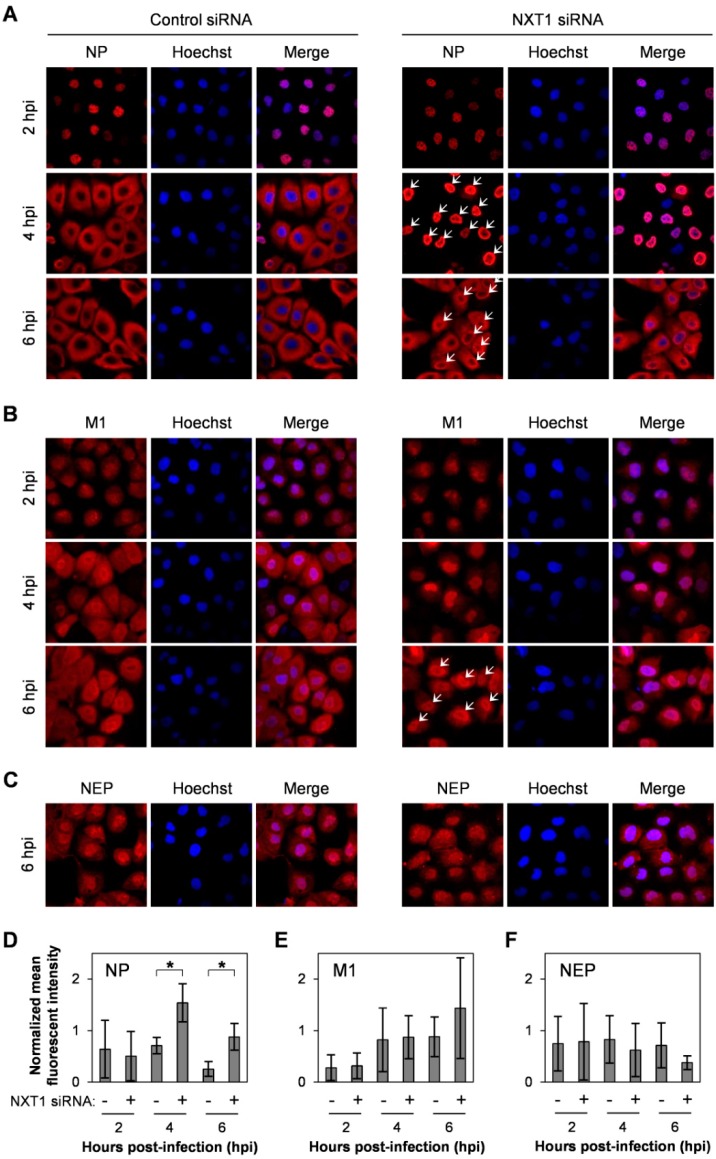
NXT1 knockdown accumulates viral NP and M1 proteins in the nucleus. (**A**–**C**) MDCK cells were transfected with control or NXT1 siRNA for 48 h and infected with influenza A/WSN/33 virus at MOI = 2. After 2, 4, or 6 hpi, the cells were analyzed by immunofluorescent staining with anti-NP (**A**); anti-M1 (**B**); or anti-NEP (**C**) antibody, followed by Alexa Fluor 594 goat anti-mouse or Alexa Fluor 594 goat anti-rabbit antibody. Nuclei were counterstained with Hoechst 33342 and the cells were visualized under an Olympus laser scanning confocal fluorescence microscope. White arrows indicate nuclear accumulation of NP or M1 proteins; (**D**–**F**) Mean fluorescence intensity of NP, M1, and NEP in the nucleus derived from (**A**–**C**) (normalized by the mean fluorescence intensity of Hoechst 33342, *n* ≥ 50). Data represent the mean ± SD values from three independent experiments. * indicates a significant difference at *p* < 0.05 (unpaired *t*-test).

**Figure 4:**
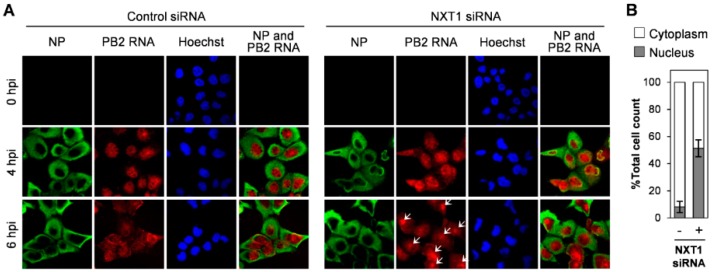
NXT1 knockdown accumulates viral genomic RNA in the nucleus. (**A**) MDCK cells were transfected with control or NXT1 siRNA for 48 h and infected with influenza A/WSN/33 virus at MOI = 10. After 4 and 6 hpi, cells were subjected to fluorescence in situ hybridization (FISH) with a PB2 segment-specific DNA probe conjugated to Quasar 670 for 16 h, followed by immunofluorescence staining with anti-NP and Alexa Fluor 488 goat anti-mouse antibodies. Nuclei were counterstained with Hoechst 33342 and cells were visualized with an Olympus laser scanning confocal fluorescence microscope. White arrows indicate accumulation of PB2 RNA in the nucleus; (**B**) %Total cell count of the cells with nuclear- or cytoplasmic-localization of PB2 RNA from (**A**) at 6 hpi (*n* ≥ 50). Data shown are the results from two independent experiments (error bars) and the mean values.

To investigate the role of NXT1 in viral replication, we monitored intracellular localization of viral proteins and vRNA in the NXT1-knockdown/infected MDCK cells at different time points post-infection. In the control siRNA-transfected cells, NP protein localized in the nucleus at 2 hpi and was mainly in the cytoplasm at 4 hpi (Figure 3A, left panel), whereas in the NXT1-knockdown cells, NP was still localized in the nucleus at 4 hpi and predominant nuclear localization at 6 hpi (Figure 3A, right panel). Viral NP protein is synthesized in the cytoplasm before being imported into the nucleus for vRNP assembly and exported to the cytoplasm for further viral particle assembly and budding. Our results clearly demonstrate that NXT1 knockdown delays nuclear export of NP, especially at 4 hpi with no cytoplasmic NP observed in the NXT-1 knockdown cells (Figure 3A,D). In addition, M1 was also slightly accumulated in the nucleus of NXT1-knockdown cells, whereas there was no effect on NEP localization (Figure 3B,C,E,F). We further investigated the effect of NXT1 on vRNA localization by FISH staining of viral PB2 RNA. In control siRNA-transfected/infected cells, PB2 RNA was detected primarily in the nucleus at 4 hpi, whereas at 6 hpi most of the PB2 RNA was localized in the cytoplasm, with some localized at the plasma membrane (Figure 4A, left panel). In contrast, PB2 RNA in 6 hpi/NXT1-knockdown cells was accumulated in the nucleus in a cluster-like pattern (Figure 4A, right panel, B). In this experiment, a higher MOI (= 10) of virus was used to facilitate visualization of the PB2 RNA, which might affect overall localization of NP. However, the pattern of NP nuclear accumulation in the 4 hpi/NXT1-knockdown cells was similar to that observed in Figure 3A, in the 6 hpi/NXT1-knockdown cells (MOI = 2). Altogether, these results suggest that NXT1 plays a role in stimulating the nuclear export of NP, M1, and vRNA to promote viral replication.

### 3.3. NXT1 Stimulates NP Nuclear Export Via a CRM1-Dependent Pathway

**Figure 5:**
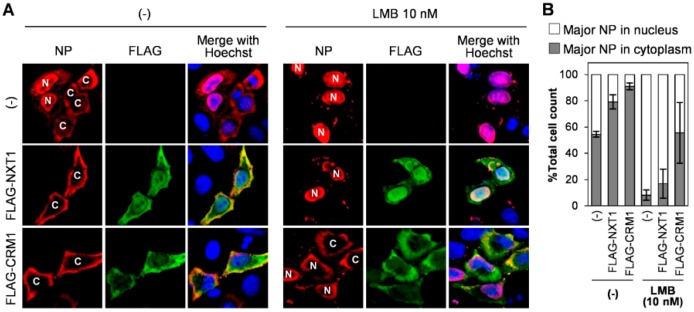
NXT1 promotes the nuclear export of NP via a chromosome region maintenance 1 (CRM1)-dependent pathway. (**A**) HeLa cells were transfected with NP/pCAGGS and FLAG-NXT1/pCAGGS or CRM1-FLAG/pCAGGS plasmids for 36 h before the addition of 10 nM leptomycin B (LMB) and cultured for 12 h. Cells were then stained with anti-NP and anti-FLAG antibodies followed by Alexa Fluor 594 goat anti-mouse and Alexa Fluor 488 goat anti-rabbit antibodies. Nuclei were counterstained with Hoechst 33342 and the cells were visualized with an Olympus confocal laser scanning microscope. N and C indicate predominant localization of NP in the nucleus and cytoplasm, respectively; (**B**) % total cell count of the cells with nuclear- or cytoplasmic-localized NP from (**A**) (*n* ≥ 50). Data shown are the results from two independent experiments (error bars) and the mean values.

We next verified the role of NXT1 in the nuclear export of NP using an NXT1 overexpression system. Transfection of NP/pCAGGS alone into HeLa cells for 48 h resulted in 55% of the cell population that exhibited NP localization in the cytoplasm (Figure 5A,B). Co-expression of NP/pCAGGS with FLAG-NXT1/pCAGGS resulted in an increased number of cells (79% of total cell count) with NP cytoplasmic localization, which was similar to the results observed following co-expression with FLAG-CRM1/pCAGGS (91% of total cell count) (Figure 5A,B). This indicates that NXT1, like CRM1, promotes nuclear export of NP. LMB, an inhibitor of CRM1, was then used to determine whether NXT1 and CRM1 interact to facilitate nuclear export of NP. LMB inhibited cytoplasmic localization of NP in NP/pCAGGS-transfected cells (Figure 5A,B). Co-transfection of FLAG-NXT1/pCAGGS with NP/pCAGGS could not reverse the effect of LMB on inhibition of nuclear export of NP, whereas NP nuclear export was partially restored in cells co-transfected with FLAG-CRM1/pCAGGS (Figure 5A,B). This result suggests that NXT1 promotes nuclear export of NP via a CRM1-dependent pathway.

### 3.4. NXT1 Forms a Complex with NP and CRM1

**Figure 6:**
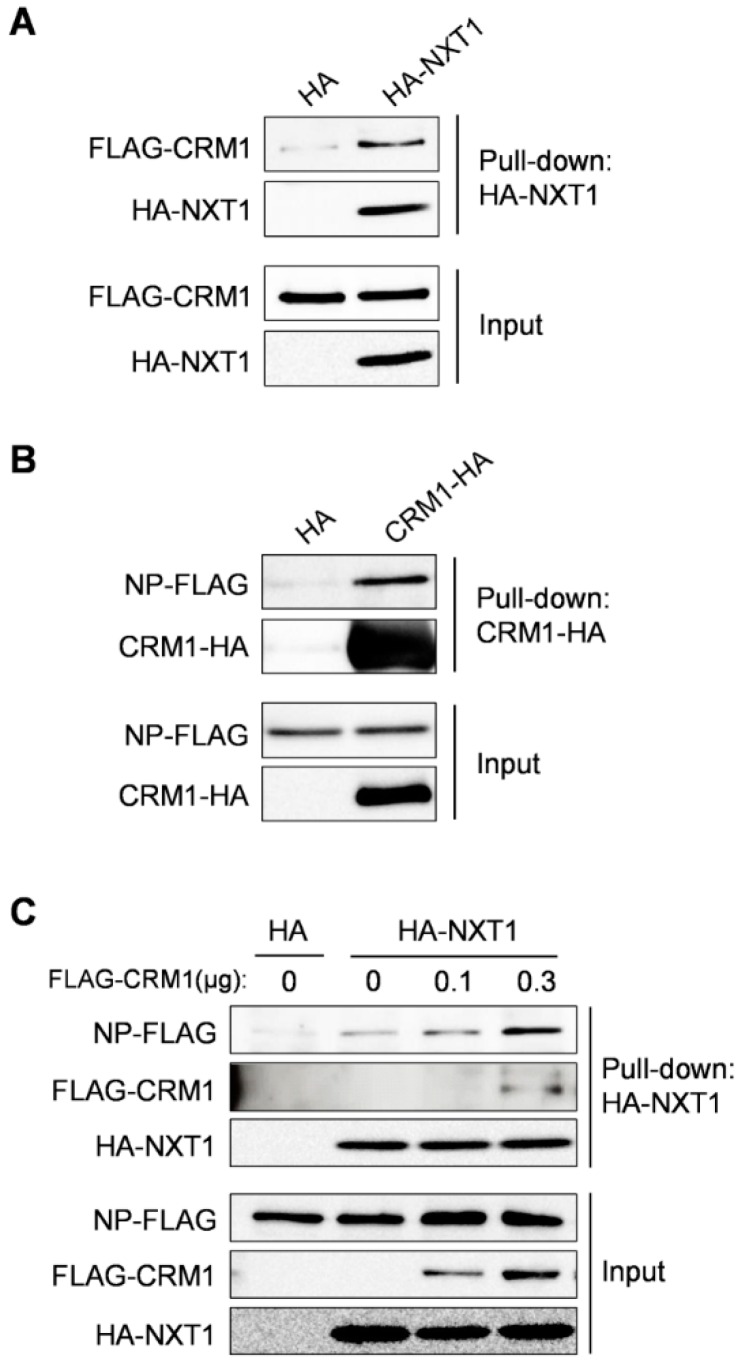
NXT1 forms a complex with NP and CRM1. HA-NXT1 or CRM1-HA protein was immobilized on anti-HA agarose beads. HA-NXT1 agarose beads were then co-incubated with purified FLAG-CRM1 protein (**A**) and CRM1-HA agarose beads were co-incubated with purified NP-FLAG protein (**B**) overnight at 4 °C. The agarose beads were collected, washed, and subjected to WB analysis with anti-FLAG and anti-HA antibodies. Data shown are the results of one representative experiment from two independent trials; and (**C**) HA-NXT1 agarose beads were co-incubated with purified NP-FLAG and different amounts of purified FLAG-CRM1 proteins. The agarose beads were collected and subjected to WB analysis with anti-FLAG and anti-HA antibodies. Data shown are the results of one representative experiment from two independent trials.

Since NP/CRM1 and NXT1/CRM1 binding have been reported [14,39] and our data in this study show an interaction between NXT1/NP, we further investigated complex formation between NP, NXT1, and CRM1 by pull-down assay. We firstly confirmed and showed the binding of NXT1/CRM1 and CRM1/NP using HA-NXT1- or CRM1-HA-immobilized agarose beads with purified FLAG-CRM1 or NP-FLAG protein, respectively (Figure 6A,B). To investigate complex formation between all three proteins, a competitive pull-down was performed by addition of FLAG-CRM1 protein to the NXT1/NP binding system. NXT1/NP binding was increased in the presence of CRM1, suggesting that CRM1 and NP did not compete with each other to bind to NXT1, but rather promoted the formation of a NXT1/NP/CRM1 complex (Figure 6C). Together, these findings suggest that NP, NXT1, and CRM1 proteins form a complex that promotes the nuclear export of NP.

### 3.5. NXT1 Interacts with the C-terminal Region of NP

**Figure 7:**
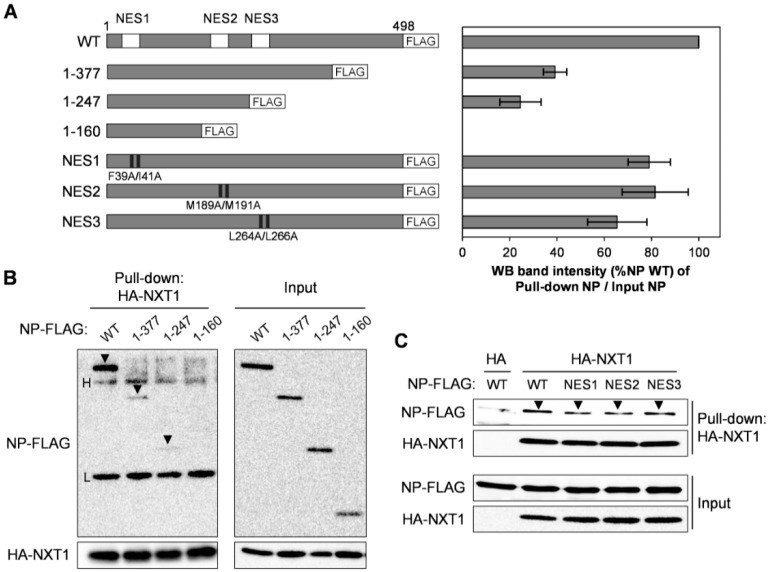
NXT1 binds to the C-terminal region of NP. (**A**) Left panel shows a schematic of NP wild-type (WT), the N-terminal partial constructs, and the NES domain mutants. Right panel shows intensity (% NP WT) of the pull-down NP band divided by the input NP band from the WB analysis of (**B**,**C**). Data shown are the results from two independent experiments (error bars) and the mean values; (**B**,**C**) HA-NXT1 protein was immobilized on anti-HA agarose beads and co-incubated overnight with purified N-terminal NP-FLAG (**B**) or NES mutant NP-FLAG (**C**). The beads were collected, washed, and subjected to WB analysis with anti-FLAG and anti-HA antibodies. Black arrowheads indicate binding of NXT1 and NP. H and L indicate the heavy and light chains of the agarose-conjugated anti-HA antibody.

To investigate the NXT1-binding site on NP protein, N-terminal NP constructs with FLAG tag including NP 1–377, NP 1–247, and NP 1–160 (Figure 7A, left panel) were used for affinity pull-down assay with HA-NXT1-immobilized agarose beads. All of the N-terminal NP constructs exhibited reduced binding to NXT1, especially the NP 1–160, which almost completely lost binding capacity (Figure 7A, right panel, B). This result indicates that the C-terminal region of NP is essential for interaction with NXT1.

NP contains three different NES domains (NES1, NES2, and NES3), of which NP-NES3 is CRM1-dependent. Mutation of the hydrophobic residues L259, L264, or L266 of NP-NES3 has been shown to partially decrease binding with CRM1 [14,40]. We further investigated whether the NP-NES domain participates in binding with NXT1 using a series of NP-NES mutants, including NP-NES1 (F39A/I41A), NP-NES2 (M189A/M191A), or NP-NES3 (L264A/L266A) (Figure 7A, left panel). No differences in the binding of the NXT1/NP-NES mutants were observed (Figure 7A, right panel, C), which suggests that the NP-NES domains are dispensable for interaction with NXT1. 

## 4. Discussion

Here, we identified NXT1 as a novel NP binding protein that stimulates the nuclear export function of influenza NP via a CRM1-dependent pathway. NXT1, which is 26% identical to NTF2, was discovered as a Ran-GTP binding protein that co-localizes with the nuclear pore complex (NPC) and shuttles between the nucleus and cytoplasm [41,42]. Later studies showed that NXT1 is a critical cofactor for TAP-mediated mRNA nuclear export by formation of a TAP/NXT1 heterodimer that enhances the TAP/RNA interaction with nucleoporins at the NPC [35,36,37]. In addition, several reports showed that NXT1 plays role in the nuclear export of different classes of RNA, such as U1 snRNA and tRNA (in a CRM1-dependent manner [38]) and intron-containing RNA (in a CRM1-independent manner [43]), as well as NES-containing proteins, such as protein kinase inhibitor (PKI) [41] and human immunodeficiency virus 1 (HIV-1) Rev (in a CRM1-dependent manner [39]). 

A role for NXT1 in CRM1-dependent nuclear export was demonstrated in digitonin-permeabilized cells by the addition of NXT1, which stimulated the nuclear export of NES-containing protein PKI and this stimulation is inhibited by LMB [41]. In addition, NXT1 is involved in the terminal step of CRM1-mediated nuclear export [39]. HIV-1 Rev is targeted to the cytoplasmic side of NPC by CRM1/Ran and requires NXT1 and RanBP1 for the terminal release step of CRM1/Rev into the cytoplasm [39]. Moreover, NXT1 directly binds to CRM1, and cells expressing the NXT1 R107A mutant, which reduces CRM1 binding, exhibit decreased nuclear export activity [39]. This mechanism may explain our data showing that NXT1 promotes nuclear export of the influenza NP protein. We showed direct binding of NP/CRM1 [14] and NXT1/NP (Figure 1, Figure 6, and Figure 7), while other studies have reported binding of NXT1/CRM1 [39]. Our results also show that binding of CRM1 and NP to the NXT1 was not competitive, and that NXT1 promoted nuclear export of NP only in the absence of LMB. Therefore, NXT1 may form a complex with CRM1/NP to stimulate nuclear export of NP. Whether this formation occurs in the nucleus or the cytoplasmic side of the NPC remains to be determined. In addition, a specific interaction of NXT1 and the C-terminal region of viral NP may be used as a target for identification of the virus-specific drug that does not affect host cell viability. 

NXT1-promoted nuclear export of NP may be involved with influenza vRNP nuclear export function. Although vRNP nuclear export was reported to depend on NEP [18], several publications argue this finding, as LMB treatment inhibits nuclear export of vRNP, but not NEP [21,44], or identify a NEP-independent vRNP nuclear export mechanism [24]. Our results show that NXT1 depletion causes nuclear accumulation of NP, M1, and vRNA, which are components of vRNP. This may suggest a role for NXT1 with CRM1, NP, and M1 in regulating vRNP nuclear export.

NXT1 and TAP-mediated nuclear export of influenza NP mRNA via association with NUP62 has been reported, and knockdown of TAP but not NXT1 decreased NP mRNA expression [26]. Our data indicates that NXT1 knockdown also does not affect the expression of NP, including HA protein, in infected cells (Figure 2B). However, M1 and NA expression are decreased, which may suggest that NXT1 selectively regulates the nuclear export of specific viral mRNA species. This is supported by the finding that individual influenza mRNAs are differentially dependent on the cellular TAP, UAP56, and Aly for nuclear export, e.g., HA mRNA is TAP-dependent; NS1 mRNA is UAP56-dependent; and M1/M2 mRNAs are TAP-/UAP56-dependent [45]. In addition, TAP/NXT1 heterodimer forms a complex with viral mRNA in vitro [26] which may involve the recruitment of mRNA to NPC for nuclear export [35,36,37]. 

In summary, we have identified a direct interaction between influenza NP and cellular NXT1 that stimulates the nuclear export of NP and vRNA to promote viral replication. NXT1-mediated nuclear export of NP occurs via a CRM1-dependent pathway. The specific binding of NP/NXT1 may be useful as a novel target for the development of antiviral drugs with minimal cytotoxic effects.
